# Phytohormonal Networks Promote Differentiation of Fiber Initials on Pre-Anthesis Cotton Ovules Grown *In Vitro* and *In Planta*

**DOI:** 10.1371/journal.pone.0125046

**Published:** 2015-04-30

**Authors:** Hee Jin Kim, Doug J. Hinchliffe, Barbara A. Triplett, Z. Jeffrey Chen, David M. Stelly, Kathleen M. Yeater, Hong S. Moon, Matthew K. Gilbert, Gregory N. Thyssen, Rickie B. Turley, David D. Fang

**Affiliations:** 1 Cotton Fiber Bioscience Research Unit, USDA-ARS-SRRC, New Orleans, Louisiana, United States of America; 2 Institute for Cellular and Molecular Biology and Center for Computational Biology and Bioinformatics, The University of Texas at Austin, Austin, Texas, United States of America; 3 Department of Soil and Crop Science, Texas A&M University, College Station, Texas, United States of America; 4 Plains Area, USDA-ARS, Fort Collins, Colorado, United States of America; 5 Crop Genetics Research Unit, USDA-ARS, Stoneville, Mississippi, United States of America; Instituto de Biología Molecular y Celular de Plantas, SPAIN

## Abstract

The number of cotton (*Gossypium* sp.) ovule epidermal cells differentiating into fiber initials is an important factor affecting cotton yield and fiber quality. Despite extensive efforts in determining the molecular mechanisms regulating fiber initial differentiation, only a few genes responsible for fiber initial differentiation have been discovered. To identify putative genes directly involved in the fiber initiation process, we used a cotton ovule culture technique that controls the timing of fiber initial differentiation by exogenous phytohormone application in combination with comparative expression analyses between wild type and three fiberless mutants. The addition of exogenous auxin and gibberellins to pre-anthesis wild type ovules that did not have visible fiber initials increased the expression of genes affecting auxin, ethylene, ABA and jasmonic acid signaling pathways within 1 h after treatment. Most transcripts expressed differentially by the phytohormone treatment *in vitro* were also differentially expressed in the ovules of wild type and fiberless mutants that were grown *in planta*. In addition to *MYB25-like*, a gene that was previously shown to be associated with the differentiation of fiber initials, several other differentially expressed genes, including *auxin/indole-3-acetic acid (AUX/IAA)* involved in auxin signaling, *ACC oxidase* involved in ethylene biosynthesis, and *abscisic acid* (ABA) *8'-hydroxylase* an enzyme that controls the rate of ABA catabolism, were co-regulated in the pre-anthesis ovules of both wild type and fiberless mutants. These results support the hypothesis that phytohormonal signaling networks regulate the temporal expression of genes responsible for differentiation of cotton fiber initials *in vitro* and *in planta*.

## Introduction

Cotton (*Gossypium* sp.) is the most commercially important natural fiber in the world [1]. Cotton fibers are single-celled trichomes that differentiate from epidermis of cotton ovules [2]. On the day of anthesis (0 days post-anthesis, DPA) 15–30% of the epidermal cells on cotton (*Gossypium hirsutum* L.) ovules differentiate into fiber initials [3,4,5]. Fiber initials are observed first at the chalazal end of the ovules on 0 DPA [3,6]. Although fiber initials at the chalazal end immediately enter into the elongation phase, new fiber initials bulge from the epidermis in a progressive wave toward the micropylar end until 4 DPA [3,7]. Cotton fiber initiation is considered to be quasi-synchronized in each developing ovule and among ovules within each ovary, and cotton ovules between 0 and 4 DPA contain a mixture of fiber initials and elongating fibers [8].

The density of fiber initials is generally considered an indicator of cotton yield [9] and a potential target for improving yield by biotechnological manipulations [5], although one claim to the contrary has been made [10]. More recent work demonstrated that a higher density of fiber initials presumably caused by higher auxin levels in the ovule epidermis at the fiber initiation stage resulted in higher fiber yield and finer fibers [11]. Thus, the ratio of fiber initials to non-fiber cells on the epidermis of cotton ovules may affect yield and cotton fiber quality.

Due to the potential for improving cotton yield and quality by increasing the number and/or density of fiber initials, several investigations have attempted to identify cotton genes that are mainly responsible for the differentiation of fiber initials from the epidermis of cotton ovules. The first transcriptome profiles compared wild type (WT) ovules at a late fiber initiation stage (3 to 5 DPA) with isogenic ovules from fuzzless and lintless (*fl*) or a naked (N_1_) mutant [12,13]. Since large portions of the differentially expressed genes (DEGs) identified in these studies overlapped with those known to be involved in fiber elongation, the second transcriptome profiles used transcripts extracted from fiber initials at an early fiber initiation stage (0 to 1 DPA) using advanced extraction techniques such as laser-capture microdissection [14] or by vortexing a mixture of frozen cotton ovules and glass beads [15]. Expression analyses of fiber initials compared with other tissues from WTs or mutants identified candidate genes involved in the fiber initiation process [14,15,16].

Among the DEGs identified by genome-wide transcriptome expression analyses were *GhMYB1-6*, *GhMyb109*, *dehydration-induced RD22-like protein* (*GhRDL*), *acetyltransferase* (*GhACY*), *fiddlehead-like protein* (*GhFDH*), *serine carboxylpeptidase protein* (*GhSCP*), *tubulins* (*GhTUA6* and *GhTUB1*), *cellulose synthase catalytic subunit* (*GhCesA5*), *actin* (*GhACT*), *sterol-C-methyltransferase* (*GhSMT*), *sucrose synthase* (*GhSuS*), *homeobox 1*, *calmodulin*, and *ER lumen protein-retaining receptor*, all of which are involved in both fiber initiation and elongation [12,13,15,17,18,19,20,21]. The other DEGs including *GhMYB25*, *GhMYB25-like*, *homeodomain-leucine zipper transcription factor* (*GhHD-1)*, *GaMYB2*, *dehydration-induced RD22-like protein 1* (*GARDL1*), *protodermal factor* (*GhPDF1*), a putative *cell wall protein* (AY464064), *fatty acid elongase* (AY464065), *lipid transfer protein* (AY464062), and a *small glycine rich protein* were proposed to be involved in fiber initiation [13,14,16,22].

Subsequent functional analyses of individual candidate genes, however, has shown that many genes identified by transcriptome profiles appear to be involved in fiber elongation rather than in the fiber initiation process. When *GbPDF1*, *GaHOX1*, *GhMYB109*, *GaRDL1*, or *GaMYb2* was individually suppressed in transgenic cotton plants, fiber length was reduced [17,22,23,24,25]. The short fiber length implies that these candidate genes are not involved in the differentiation of fiber initials from ovular epidermal cells but may be involved in early fiber development. None of these candidate genes were highly expressed at 0 DPA when the fiber initials were first detected in the ovules. Transcript abundance increased from 0 to 3–5 DPA and then decreased. RNAi-mediated suppression of *GhMYB25* slightly reduces the number of fiber initials but markedly reduces fiber length [26]. Ectopic overexpression of *GhMYB25* increases the number of fiber initials and leaf trichome number. The phenotypes of transgenic cotton plants with suppressed levels of *MYB25*, *MYB109*, or *PDF1* indicate that these candidate genes may play roles in fiber elongation although they were originally indentified as genes involved in fiber initiation.

Among numerous candidate genes identified by extensive transcriptome profiles, two transcription factors, GhMYB25-like [27] and GhHD-1 [28], have been verified to be involved in differentiation of fiber initials from the cotton ovule epidermis. Suppression of *GhMYB25-like* expression resulted in no differentiation of fiber initials on cotton ovules, yet overexpression did not increase the number of fiber initials, suggesting that other factors interacting with GhMYB25-like may be required for fiber initial differentiation. Suppression of *GhHD-1*, a homeodomain-leucine zipper transcription factor that is structurally similar to the *Arabidopsis* GLABRA2 transcription factor (GL2) involved in leaf trichome differentiation, delayed the timing of fiber initiation. Overexpression of *GhHD-1* increased the number of fiber initials on the seed without affecting leaf trichomes [28]. In addition, functional analyses with transgenic cottons have shown that two enzymes, sucrose synthase (GhSus) [18,29] and a vacuolar invertase (GhVIN 1) [20,21], are involved in both fiber initiation and elongation.

Despite the recent progress in identifying two transcription factors, GhMYB25-like and GhHD-1, responsible for the differentiation of fiber initials from epidermis, the molecular mechanisms regulating the cotton fiber initiation process are mostly unknown. In the model plant *Arabidopsis*, the processes regulating the differentiation of leaf trichomes and root hairs from epidermal cells have been well characterized, and the number and the types of regulatory interactions between multiple transcription factors are complex [30,31]. Some common transcription factors such as GL2, GL3/EGL3, and TTG1 regulate the differentiation of both trichomes and root hair cells from epidermal cells; however, these processes are regulated by different phytohormones [30]. The number and density of trichome cells in *Arabidopsis* can be increased by treatments with gibberellin acid (GA), cytokinins, and jasmonic acid, and decreased by salicylic acid treatment. In contrast, root hair formation in *Arabidopsis* is promoted by auxin and ethylene.

Cotton fiber differentiation from ovule epidermal cells is likely regulated by multiple transcription factors that are regulated by phytohormones as the case in *Arabidopsis*. Indeed, unknown cotton proteins were reported to interact with GhMYB25-like transcription factor [27]. Cotton expressed sequence tag (EST) analyses also showed that MYB and WRKY transcription factors predicted to be involved in auxin, GA, ethylene, brassinosteroid (BR), and abscisic acid (ABA) signaling pathways were more enriched in cotton ovules at the fiber initiation stage (-3, 0, and +3 DPA) than in other non-fiber tissues [32]. Auxin and GA are essential for differentiating fiber initials *in vitro* [33]. Although there is a general agreement that multiple networks of phytohormonal regulators and transcription factors are required for differentiating fiber initials, only very limited numbers of the candidate genes have been verified [12,13,14,15,16,34].

Based on the temporal and spatial expression patterns of *GhMYB25-like* and *GhHD-1*, their indispensable roles in the differentiation of fiber initials and their up-regulation in pre-anthesis ovules (-2 to -1 DPA) containing no fiber initials [27,28], we hypothesize that pre-anthesis cotton ovules are enriched with the transcripts required for fiber differentiation.

In this paper, we used comparative transcript profiling to identify genes whose expression changed after the exogenous application of auxin and GA to pre-anthesis cotton ovules grown *in vitro*. Candidate DEGs identified from in cultured cotton ovules were verified as important for fiber initiation by measuring the transcript abundance of candidate genes in the ovules of three fiberless (*fl*) mutants compared with a WT grown *in planta*. Our results support the hypothesis that phytohormonal signaling networks and crosstalk regulate the temporal expression of genes responsible for the differentiation of fiber initials *in vitro* and *in planta*.

## Materials and Methods

### Plant material

*G*. *hirsutum* WT lines (TM-1 and Xu-142) and three fiberless mutant lines (Xu-142 *fl*, MD17, and SL-1-7-1) were grown in a field at the USDA-ARS, Southern Regional Research Center in New Orleans, LA. Standard growing practices were applied during the growing season. The soil type in New Orleans was aquent dredged-over alluvium in an elevated location to provide adequate drainage. Two biological replications (6 bolls per replicate) of unfertilized ovules from cotton squares (TM-1, -1 DPA) were collected for cotton ovule culture. For DEG validation, three biological replications (4 bolls per replicate) of cotton ovules (-3, -1, 0, 1, and 3 DPA) from a WT (Xu-142) and the three fiberless mutant lines were harvested. All ovules were collected in the morning between 0800 and 0900. The collected ovules were either frozen with liquid nitrogen for RNA extraction and qPCR analyses or fixed for microscopic analyses.

### Cotton ovule culture

Cotton ovaries harvested from the cotton squares (-1 DPA) were surface-sterilized and dissected under sterile conditions. The unfertilized ovules were transferred to a liquid basal medium in the presence or absence of 5μM indole-3-acetic acid (IAA) and 1μM GA and incubated at 32°C in a 5% CO_2_ atmosphere [33,35]. Cotton ovules incubated for 1, 3, 6, 12, and 24 h were harvested for RNA extraction, microarray and image analyses.

### Scanning electron microscopy

Cotton ovules were fixed with 3% (v/v) glutaraldehyde in 0.1 M sodium phosphate, pH 7.0, dehydrated in a graded ethanol series starting from 20% (v/v) up to 100% (v/v) ethanol and mounted on standard Cambridge SEM stubs. SEM images were taken with a XL30 Environmental Scanning Electron Microscope (FEI Company, Hillsboro, OR) at an accelerating voltage from 10–15 kV under high vacuum conditions.

### RNA extraction from cotton fibers

Total RNA was extracted from the frozen ovules using a Sigma Spectrum Plant Total RNA Kit (Sigma-Aldrich, St. Louis, MO) with DNase1 digestion according to the manufacturer’s protocol. The quality and quantity of total RNAs were determined using a NanoDrop 2000 spectrophotometer (NanoDrop Technologies Inc., Wilmington, DE) and an Agilent Bioanalyzer 2100 (Agilent Technologies Inc., Santa Clara, CA).

### Microarray hybridizations and data analysis

Cotton oligonucleotide arrays version 2 [36] representing 22,787 cotton probes designed from *Gossypium* expressed sequence tag (EST) sequences were used to compare the expression of genes in cultured ovules (-1 DPA) treated with or without phytohormones (5μM IAA and 1μM GA). The phytohormone incubation periods for -1 DPA unfertilized ovules were 0, 1, 3, 6, and 12 h with two biological replicates at each time-point. Procedures for RNA amplification, labeling with Cy5 or Cy3, hybridization, scanning, data normalization and assessment of statistically and biologically significant genes were performed as described previously [37]. Statistical analyses were performed using JMP Genomics version 3.2 (SAS Institute Inc., Cary, NC, USA) to identify significant DEGs (fold change > 2) in fibers of each cotton NIL at each time point with *p*-value ≤ 0.05. Primary annotation was performed using Blast2GO [58], and blastx in The *Arabidopsis* Information Resource (http:/www.arabidopsis.org/Blast/) and *Gossypium raimondi*i genome sequence (http:/www.phytozome.net/cotton.php). Gene Ontology (GO) enrichment and visualization of microarray results were performed according to the described method [38]. For the singular enrichment analysis, the identified DEGs were compared with the *Gossypium raimondii* genome [39]. For the sequence analyses, *Arabidopsis* orthologues of the identified DEGs from the cotton ovules were compared with *Arabidopsis* genome sequences (http:/www.arabidopsis.org/index.jsp). For statistical analyses and GO enrichment, the *p*-value cutoff for significance was 0.05. The GEO accession number was assigned as GSE66251.

### Reverse transcription quantitative PCR (RT-qPCR)

The experimental procedures and data analysis related to RT-qPCR were performed according to the Minimum Information for Publication of Quantitative Real-Time PCR Experiments (MIQE) guidelines [61]. The cDNA synthesis reactions were performed using the iScript cDNA Synthesis Kit (Bio-Rad Laboratories, Hercules, CA) according to the manufacturer’s instructions with 1 μg of total RNA per reaction used as template. Control cDNA synthesis reactions to check for genomic DNA contamination during RT-qPCR consisted of the same template and components as the experimental reactions without the reverse transcriptase enzyme. Thirty-seven pairs of specific primers were designed from twenty-nine DEGs for validation of the microarray results. The RT-qPCR reactions used iTaq SYBR Green Supermix (Bio-Rad Laboratories) in a Bio-Rad CFX96 real time PCR detection system. Thermal cycler parameters for RT-qPCR were as follows: 95°C 3 minutes, 50 cycles of 95°C 15 seconds, 60°C 30 seconds. A dissociation curve was generated and used to validate that a single amplicon was present for each RT-qPCR reaction. The calculations for amplification efficiencies of the target and reference genes and the relative quantifications of the different target gene transcript abundance were performed using the comparative Cq method as described [61] with the following modification: the average of three reference gene Cq values was determined by taking the geometric mean which was used to calculate the ΔCq values for the individual target genes. The endogenous reference genes used in the RT-qPCR reactions were the *18S rRNA* (U42827), *ubiquitin-conjugating protein* (AI730710), and *α-tubulin 4* (AF106570). The reference and target gene primer sequences are shown in S1 Table. Three biological replications and five technical replications were used for each time-point sample.

## Results

### Differentiation of fiber initials on pre-anthesis cotton ovules grown *in planta*

**Fig 1:**
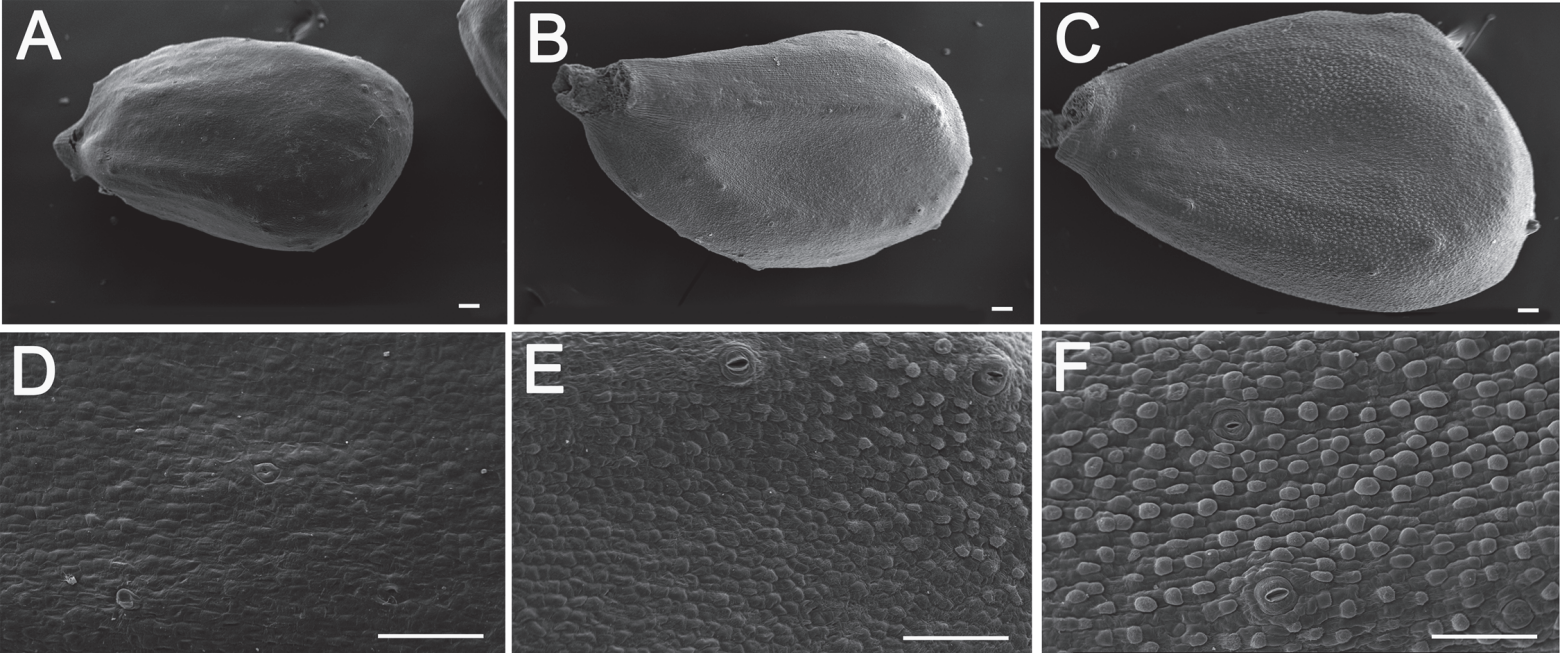
Fiber initiation of *G*. *hirsutum* grown *in planta*. Scanning microscopic images of *G*. *hirsutum* TM-1 unfertilized ovules (top panel) and epidermal tissue (bottom panel) were photographed from field-grown ovules that were harvested at 0900 on -1 DPA (A & D) and 1700 on -1 DPA (B & E), and 0800 on 0 DPA (C & F). Scale bars equal 50μm.

To determine when and how fiber initials are differentiated on cotton ovules (*G*. *hirsutum*, TM-1) grown in our field conditions, we obtained scanning electron microscopic (SEM) images from unfertilized Upland cotton TM-1 ovules harvested from field grown plants at two different time points (-1 and 0 DPA). There were no visible fiber initials on unfertilized ovules harvested at -1DPA (0900) (Fig 1A and 1D), whereas visible fiber initials were evident at very top of the chalazal end by 1700 on the same day (Fig 1B and 1E). Unfertilized ovules harvested at 0800 on 0 DPA had fiber initials covering most of the ovular surface except the micropylar ends (Fig 1C and 1F).

### Differentiation of fiber initials on pre-anthesis ovules grown *in vitro* with exogenous phytohormones

**Fig 2:**
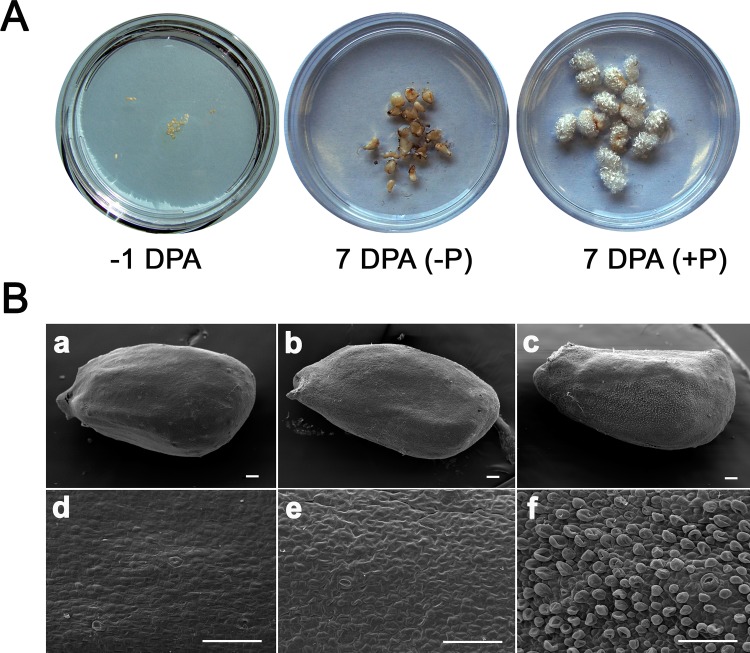
Fiber initiation of *G*. *hirsutum* grown *in vitro*. **A.** Phytohormonal effects on fiber development in cultured ovules. One day before anthesis (-1 DPA) ovules (*G*. *hirsutum*, TM-1) containing no fiber initials were treated with exogenous phytohormones (+P) consisting of 5μM IAA and 1μM GA until 7 DPA. No phytohormone (-P) was used as a negative control. **B.** Differentiation of fiber initials cultured with exogenous phytohormones. Scanning microscopic images of *G*. *hirsutum* TM-1 unfertilized ovules (top panel) and epidermal tissue (bottom panel) were photographed before (a & d) and after culture for 24 h on basal medium either in the absence (b & e) or presence (c & f) of phytohormones (5μM IAA and 1μM GA). The bars represent lengths of 50μm.

Since no fiber initials had differentiated before 0900 on -1 DPA, we tested whether the exogenous application of auxin and GA could promote fiber initiation *in vitro*. When the -1 DPA ovules were cultured with 5μM IAA and 1μM GA for 8 days, the 7 DPA ovules were covered with developing fibers (Fig 2A). In contrast, no visible fibers were detected on the 7 DPA ovules cultured in the absence of phytohormones, although ovule size substantially increased as compared with those at -1 DPA.

Fig 2B showed that the phytohormone treatment differentiated fiber initials from the -1 DPA ovules within 24 hours. The unfertilized -1 DPA ovules used for the culture contained no fiber initials on the surface (Fig 2B, a & d). Cotton ovules cultured for 24h without the phytohormones maintained the same ovule surface containing no fiber initials (Fig 2B, b & e), whereas the ovules cultured for 24h with the phytohormones differentiated fiber initials on the surface of the ovule (Fig 2B, c & f).

### Transcriptome profiles of pre-anthesis ovules grown *in vitro* with exogenous phytohormones

**Fig 3:**
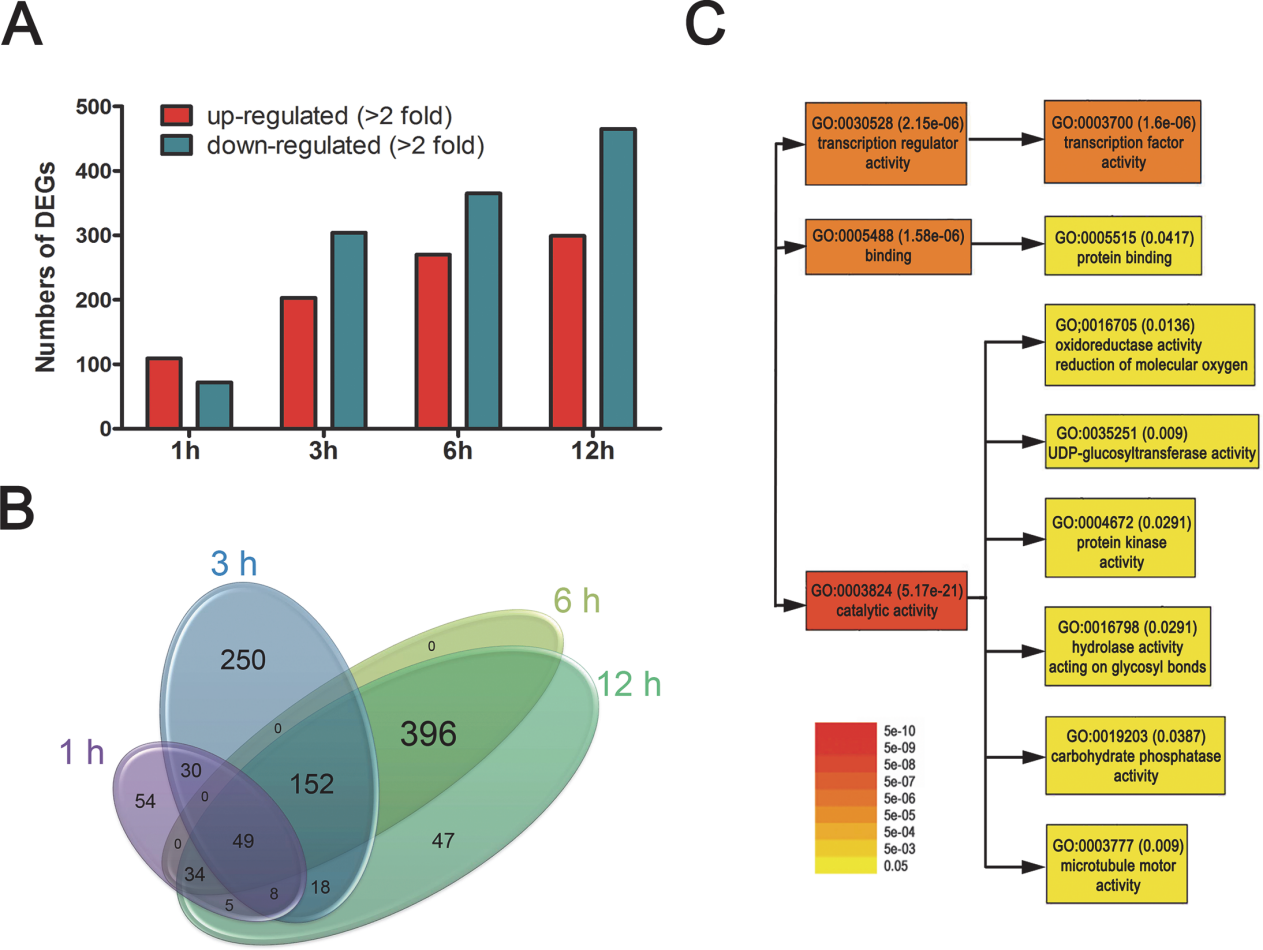
Summary of microarray analysis of cotton ovules cultured with phytohormones. **A.** Comparison of up- or down-regulated DEGs in cultured cotton ovules (-1 DPA) that were treated for 1, 3, 6, and 12 hours with IAA + GA. **B.** Venn diagrams representing the common and specific DEGs among the four time points in cultured cotton ovules (-1 DPA). **C.** GO enrichment analysis. Singular enrichment analysis was used to identify GO categories potentially governing differential expression of genes involved in fiber initiation caused by exogenous phytohormone treatment. The color and numbers adjacent to the GO identifier represent p-values.

Transcriptome profiles were obtained from ovules harvested before 0900 on -1 DPA and treated with phytohormones (5μM IAA and 1μM GA) for 1, 3, 6 and 12 hours in ovule cultures. Ovule cultures with no phytohormone treatment served as the control. Among the 22,787 probes contained on the cotton oligonucleotide array chip, 1,043 unique transcripts were differentially expressed with more than a 2-fold difference (S1 File). As the incubation period with the phytohormones increased from 1, 3, 6, to 12 h, the numbers of DEGs increased from 181, to 507, to 631, to 764, respectively (Fig 3A). Comparisons of the DEGs at the four different time points showed three distinctive patterns of temporal regulation (Fig 3B). Of the DEGs, 54, 250, and 396 DEGs specifically identified at 1, 3h and both 6 and 12h, respectively appear to be involved in immediate, intermediate, and late responses to the exogenous phytohormones.

### Gene ontology analyses of differentially expressed genes in cultured ovules

To identify the potential processes governing the 1,043 DEGs that may be involved in the differentiation of fiber initials by phytohormones, singular enrichment analysis was used from agriGO [38]. Significantly enriched (*p*-value ≤ 0.05) genes included those encoding proteins with transcription regulator activity, protein-binding activity, and catalytic activities (Fig 3C). The catalytic activities found among the DEGs included oxidoreductases, UDP-glucosyltransferases, kinases, hydrolases, and phosphatases. Transcription factors such as *IAA29*, *IAA11*, *IAA7*, *IAA16*, *IAA4*, and *ARF16* that are involved in auxin signaling pathways in *Arabidopsis* were mainly up-regulated within 1h (Table 1). Among them, *IAA29* was the most up-regulated (1,673 fold at 3 h) by the exogenous phytohormone treatment (Table 1). A WRKY23 is a transcription factor that responds to auxin and nematodes in *Arabidopsis*. In addition, *MYB* and *TCP transcription factors* [40,41] known to be involved in fiber initiation and elongation in cotton were also up-regulated within a 1 h treatment (Table 1). The GhHD-1 transcription factor (Gorai.013G167800) proposed to be involved in fiber initiation was up-regulated after 3 or 6 h of phytohormone treatment (S1 File).

**Table 1:** Transcription factors regulated in pre-anthesis cotton ovules (-1 DPA) by auxin and GA.

No	Transcriptional factor names	*G*. *raimondii* ID	Cotton EST ID	TAIR ID	Array transcript levels				
					0h	1h	3h	6h	12h
**1**	indole-3-acetic acid inducible 29 (IAA 29)	Gorai.001G043900	CGI8_TC65241;	At4g32280	1.0	100.3	1673.0	147.9	36.1
			Cotton12_11151_01						
**2**	bHLH DNA-binding protein	Gorai.001G103300	Cotton12_18505_01	At3g07340	1.0	9.8	-	-	1.3
**3**	GATA transcription factor 26 (GATA26)	Gorai.006G214400	Cotton12_17078_01	At4g17570	1.0	7.5	5.1	3.1	0.8
**4**	High mobility group B2	Gorai.002G115100	AI054798	At1g20693	1.0	6.5	2.1	1.3	1.5
**5**	MYB	Gorai.013G196800	CGI8_TC75953	At1g08810	1.0	5.3	8.2	3.0	3.6
**6**	C2H2 type zinc finger protein	Gorai.013G155400	Cotton12_05478_01	At4g27240	1.0	5.0	53.3	1.3	1.1
**7**	indole-3-acetic acid inducible 11 (IAA 11)	Gorai.007G150000	Cotton12_21593_01;	At4g28640	1.0	3.8	2.7	2.4	2.7
			Cotton12_15680_01						
**8**	auxin-responsive protein iaa7 (IAA 7)	Gorai.009G132200	Cotton12_AJ458442;	At3g04730	1.0	3.1	3.7	2.8	2.4
			Cotton12_16710_01						
**9**	B-box type zinc finger protein	Gorai.004G113000	Cotton12_15879_01	At1g25440	1.0	2.7	1.2	12.0	2.2
**10**	Indole acetic acid-induced protein 16 (IAA 16)	Gorai.007G276900	CGI8_TC72612;	At3g04730	1.0	2.4	3.1	3.2	1.0
			Cotton12_00037_06						
**11**	TCP transcription factor	Gorai.012G166500	Cotton12_03262_01	At1g58100	1.0	2.2	3.1	2.2	2.2
**12**	NF-YB8, nuclear factor Y, subunit B8	Gorai.011G241600	Cotton12_06501_01	At2g37060	1.0	2.2	11.5	3.4	1.2
**13**	AUX2-11 (IAA4)	Gorai.007G277000	Cotton12_08080_01	At5g43700	1.0	2.0	2.1	2.5	1.3
**14**	WRKY23 DNA-binding protein	Gorai.006G265200	Cotton12_10616_01	At2g47260	1.0	1.7	2.3	0.6	1.8
**15**	LHW transcription factor	Gorai.009G082800	Cotton12_21049_01	At2g27230	1.0	1.1	0.2	0.2	0.0
**16**	C2H2-like zinc finger	Gorai.007G002600	CGI8_TC79812	At2g01940	1.0	1.0	6.8	0.8	0.1

In contrast, few genes associated with GA signaling pathways were identified among the DEGs. The genes for *gibberellin 20-oxidase* and *gibberellin 3 β-hydroxylase*, two GA biosynthetic enzymes, were down-regulated when the ovules were incubated with auxin and GA for 12 h (Table 2). Interestingly, the exogenous application of auxin and GA affected the expression levels of genes involved in other phytohormone signaling pathways such as ethylene, abscisic acid (ABA), and jasmonic acid (JA). 1-Aminocyclopropane-1-carboxylate (ACC) oxidase, a key enzyme for ethylene biosynthesis [42], was up-regulated at 1h, and then transcript abundance gradually decreased. ABA 8'-hydroxylase, involved in ABA degradation [43], was up-regulated at 1h and continued to be highly expressed up to 12 h (Table 2).

**Table 2:** Phytohormonal pathways regulated in pre-anthesis cotton ovules (-1 DPA) by auxin and GA.

No	Phytohormone signaling pathway	*G*. *raimondii* ID	Cotton EST ID	TAIR ID	Array transcript levels				
					0h	1h	3h	6h	12h
**GA signaling pathway**									
1	gibberellin 20-oxidase	Gorai.006G005500	CGI8_TC64916	At5g51810	0.99	1.17	1.06	1.40	0.33
2	gibberellin 3 β-hydroxylase	Gorai.010G227700	CGI8_TC73128	At1g15550	1.00	1.03	1.01	0.43	0.35
**Ethylene signaling pathway**									
3	ACC oxidase	Gorai.001G011100	Cotton12_11109_01	At2g19590	1.00	5.41	2.06	0.17	0.71
			Cotton12_34165_01	At2g19590	1.00	3.62	0.71	0.07	0.27
4	ACC oxidase	Gorai.009G182300	Cotton12_04011_01	At1g05010	1.00	1.42	0.97	0.46	0.15
**ABA signaling pathway**									
5	ABA 8'-hydroxylase	Gorai.004G177200	CGI8_TC80236	At4g19230	1.00	2.11	2.26	-	1.83
**Jasmonic acid/ H**_**2**_ **O**_**2**_ **common pathway**									
6	proline dehydrogenase	Gorai.007G186600	Cotton12_CD485620	At5g38710	1.00	1.05	3.36	1.95	0.96
7	WRKY33	Gorai.012G119600	Cotton12_AJ458442	At2g38470	1.00	3.14	3.70	2.78	2.36
8	jasmonate resistant 1 (JAR 1)	Gorai.001G156600	CGI8_TC78256	At2g46370	1.00	1.34	0.30	0.53	0.10

### Validation of differentially expressed genes in cultured ovules

**Fig 4:**
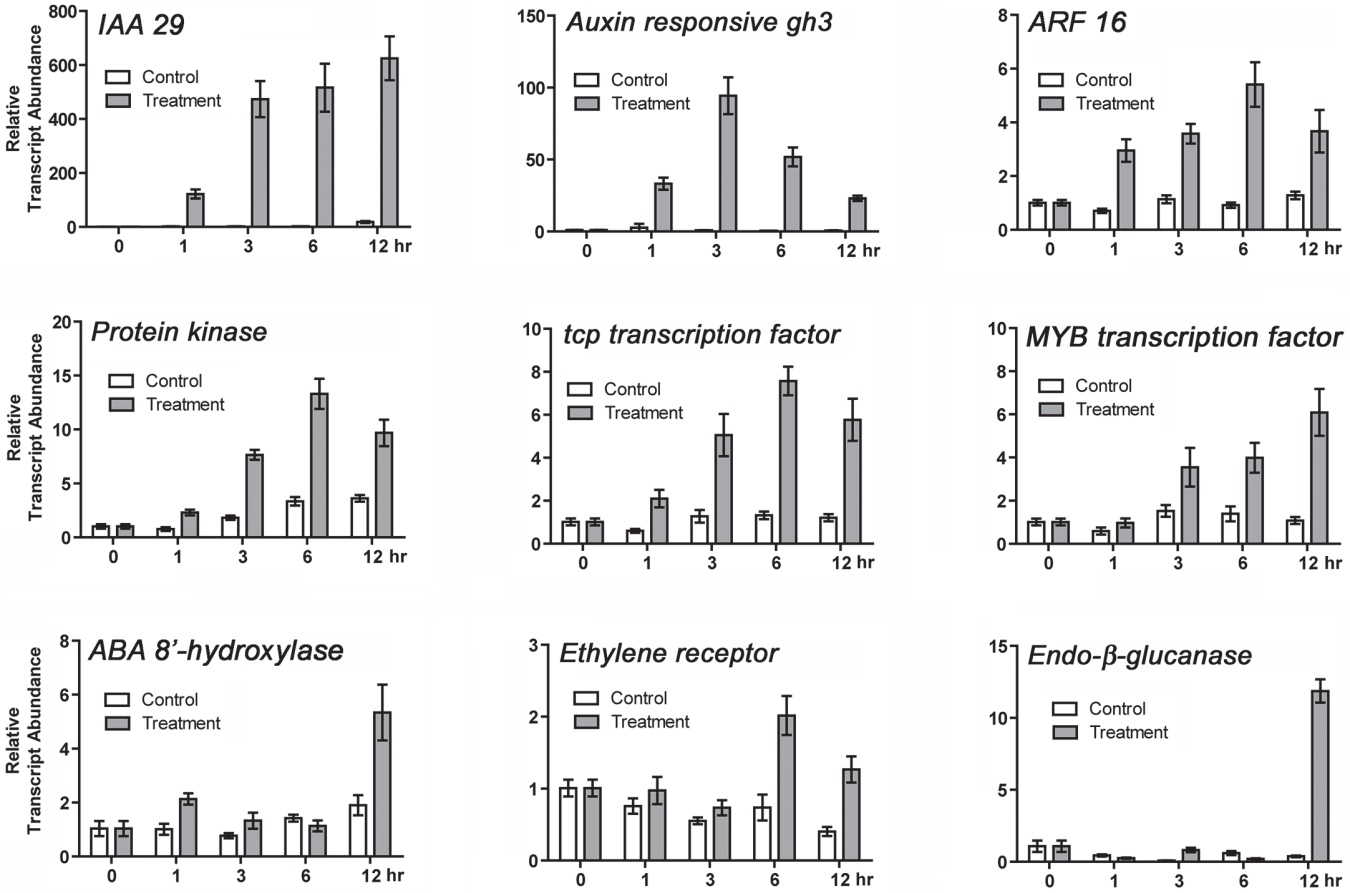
Validation of array data by RT-qPCR analysis. Genes differentially expressed due to exogenous auxin and GA treatment of unfertilized cotton ovules (-1 DPA) for 0, 1, 3, 6, and 12 h included: *IAA 29* (Gorai.001G043900)), *auxin responsive gh3* (Gorai.005G234200), *ARF 16* (Gorai.011G238900), *protein kinase* (Gorai.008G294700), *tcp transcription factor* (Gorai.012G166500), *MYB transcription factor* (Gorai.013G196800), *ABA 8'-hydroylase* (Gorai.004G177200), *ethylene receptor* (Gorai.002G038300), *endo-β -glucanase* (Gorai.007G126900). All RT-qPCR analyses were performed with three biological replications for each time point with five technical replications. The error bars represent the SD.

The DEGs identified by the arrays were validated with reverse transcription quantitative PCR analysis (RT-qPCR). Consistent with the array results (Table 1 and S1 File), the *IAA 29 transcription factor* belonging to an auxin/indole-3-acetic acid-responsive (AUX/IAA) gene family was induced by the exogenous phytohormone treatment within 1 hr and continued to be highly expressed for 12 h (Fig 4). The expression patterns of other auxin signaling pathway-related transcripts such as *auxin responsive gh3*, *ARF 16* and *protein kinase* were similar to that of *IAA 29*. The expression of *tcp transcription factor* and *MYB transcription factor* involved in fiber development [35, 36] was also up-regulated by the phytohormones. *ABA 8´-hydroxylase*, a key enzyme for ABA catabolism, was slightly induced after a 1h treatment with auxin and GA and up-regulated more significantly at 12 h. An *ethylene receptor* was up-regulated after 6 h. *Endo-β-glucanase* whose transcripts were highly expressed in actively elongating fibers [44] was induced after 12 h of phytohormone treatment.

### Phenotypes of wild type and three fiberless cotton mutants *in planta*

**Fig 5:**
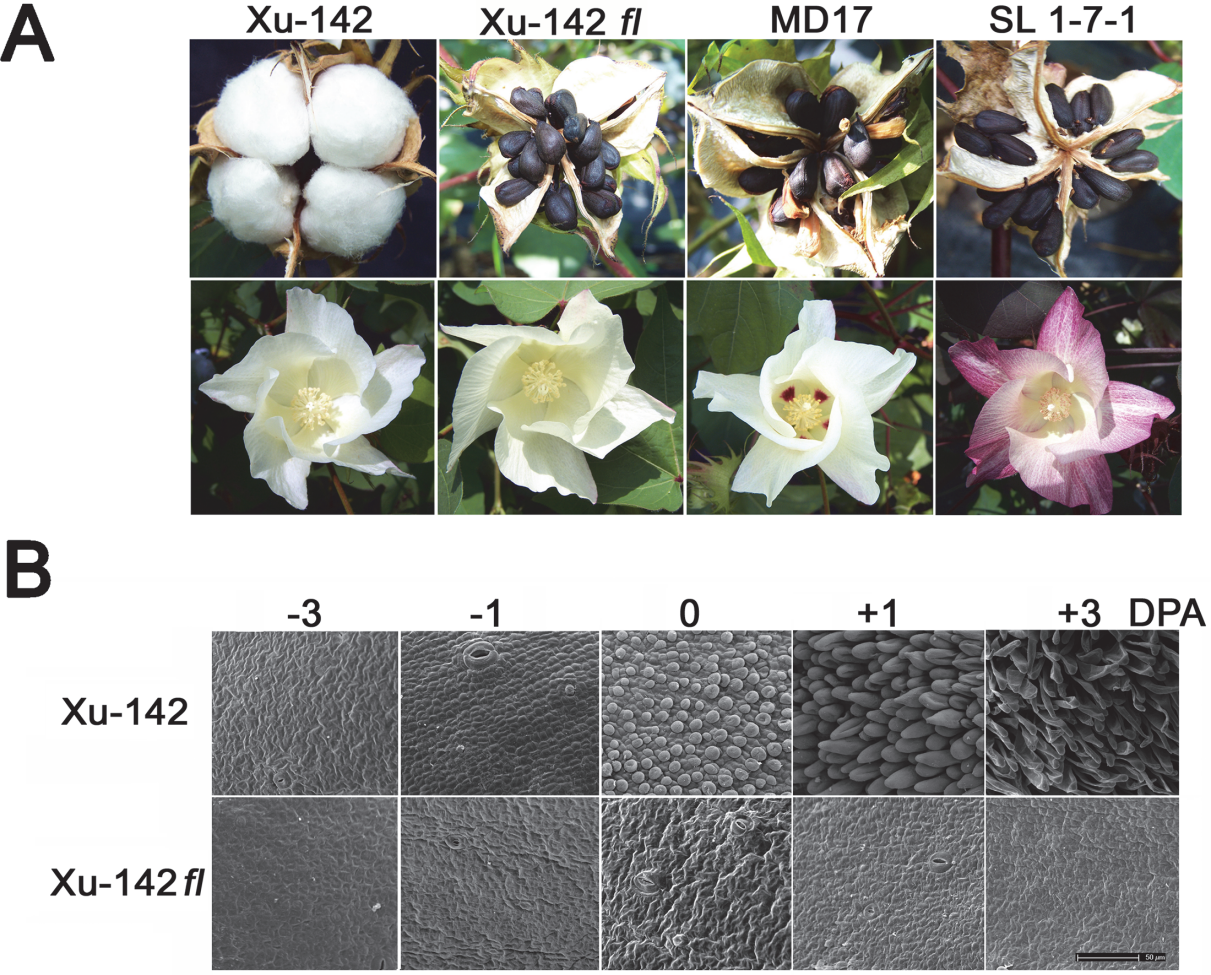
Phenotypic comparisons of a wild type and three different fiberless cotton mutants. A. Cotton bolls (top panel) and flowers (bottom panel) are from WT (Xu-142) and three fiberless mutants (Xu-142 *fl*, MD 17, and SL 1-7-1). B. SEM images of epidermal tissue from *G*. *hirsutum* WT Xu-142 ovules (top panel) and fiberless Xu-142 *fl* mutant ovules (bottom panel). The ovules were harvested around 0900 on -3, -1, 0, +1, and +3 DPA. The bar represents the length of 50μm.

To determine if the DEGs identified from the cultured ovules differentiating fiber initials by exogenous phytohormones are really involved in fiber differentiation *in planta*, we used a fuzzless and lintless (*fl*) Xu-142 *fl* mutant and its isogenic WT Xu-142 that have been extensively used to study fiber initiation [45]. In addition, two other fiberless mutants, MD17 and SL1-7-1 that have noticeable variances in flower phenotypes from Xu-142 *fl* were also used [46,47] (Fig 5A). The SEM images of the cotton ovule surface showed that fiber initials had differentiated between -1 and 0 DPA on the WT cotton ovules and the initials had elongated from 0 to 3 DPA, whereas no fiber initials had differentiated on ovules (-3 to 3 DPA) of the Xu-142 *fl* mutant (Fig 5B). Like the Xu-142 *fl* mutant, the two other fiberless mutants had no fiber initials on the 0 DPA ovules when they were photographed by our group (S1 Fig 1) as well as others [16,48].

### Temporal expression of the DEGs between wild type and three fiberless mutants *in planta*

The comparative transcriptome analyses of the cultured cotton ovules in which fiber initial differentiation is triggered by exogenous phytohormones was enriched with putative genes involved in fiber initiation. Among the DEGs identified by the transcriptome analyses, some genes may be involved in ovule development alone, whereas other genes may be regulated by phytohormones but are not involved in fiber and ovule development. To identify authentic DEGs involved in the differentiation of fiber initials *in planta* as well as *in vitro*, we compared the temporal expression of the DEGs between WT and the three fiberless mutants grown *in planta* by RT-qPCR. Based on their temporal expression in cotton ovules during the initiation stage of fiber development (-3 to +3 DPA) between the WT and three fiberless mutants grown *in planta*, the expression patterns of the DEGs were classified into three different classes.

**Fig 6:**
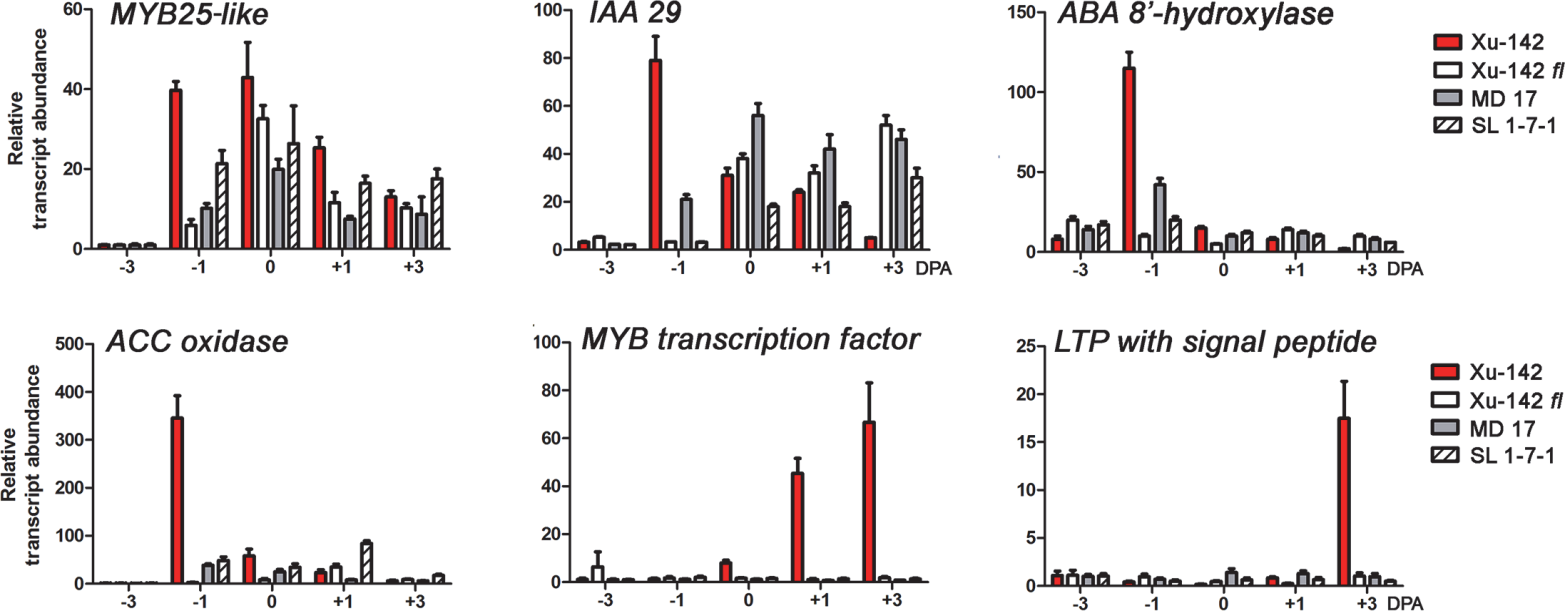
Expression patterns of Class 1 DEGs, genes more highly expressed in wild type ovules than the fiberless mutant ovules. Transcript levels were measured by RT-qPCR from a WT (Xu-142) and three fiberless mutants (Xu-142 *fl*, MD 17, and SL 1-7-1) that were harvested on -3, -1, 0, 1, and 3 DPA. *MYB25-like* (Gorai.008G179600), *IAA 29* (Gorai.001G043900)), *ABA 8'-hydroylase* (Gorai.004G177200), *ACC oxidase* (Gorai.001G011100), *MYB transcription factor* (Gorai.013G196800), *LTP with a signal peptide* (Gorai.008G057100). All RT-qPCR analyses were performed with three biological replications at each time point with five technical replications. The error bars represent the SD.

Class 1 DEGs, “DEGs more highly expressed in the WT than in the fiberless mutants”, were temporarily and specifically expressed in initiating and/or elongating fibers grown *in planta* as well as *in vitro*. Among them, three DEGs *IAA 29*, *ACC oxidase*, and *ABA 8'-hydroxylase* that were up-regulated in cultured ovules by 1h phytohormone treatment were identically co-regulated with *MYB25-like* known to be involved in the differentiation of fiber initials [27]. They were all up-regulated specifically at -1 DPA WT ovules over the three fiberless mutants (Fig 6). The temporal expression pattern for *IAA29*, *ACC oxidase*, and *ABA 8'-hydroxylase*, namely higher expression at -1DPA in WT ovules compared with the three fiberless mutants, suggests that these genes may be involved in the differentiation of fiber initials from cotton ovules as was documented for *MYB25-like*. Up-regulated expression of a *MYB transcription factor* in WT ovules occurred on 0 DPA and increased until 3 DPA. As a *lipid transfer protein* (*LTP*) was up-regulated in cultured ovules by the 12 h exogenous phytohormone treatment, the expression of both an *LTP* containing a signal peptide (TC275175) and another *LTP* missing a signal peptide (TC275775) began to be up-regulated at 3 DPA in WT ovules *in planta* (Fig 6).

**Fig 7:**
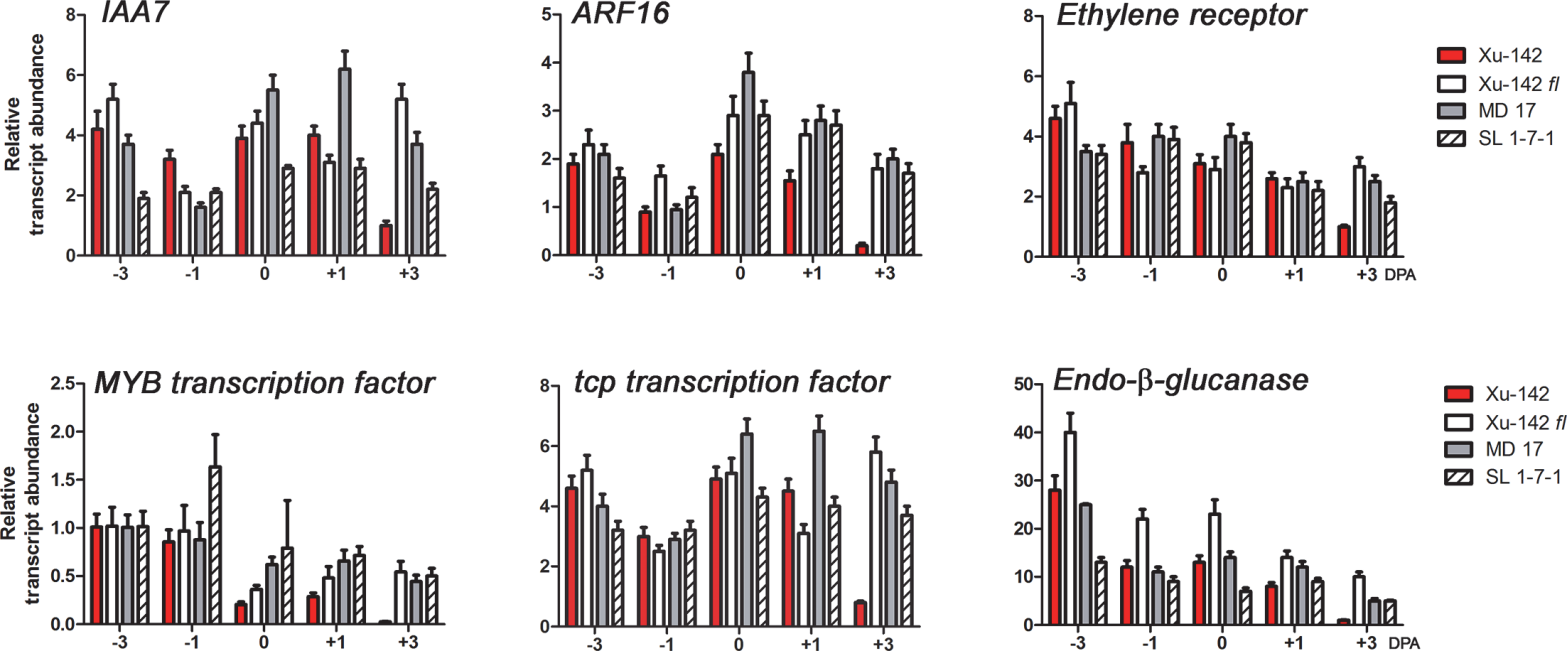
Expression patterns of Class 2 DEGs, genes more highly expressed in fiberless mutant ovules than wild type ovules at 3DPA. Transcript levels were measured by RT-qPCR from a WT (Xu-142) and three fiberless mutants (Xu-142 *fl*, MD 17, and SL 1-7-1) that were harvested on -3, -1, 0, 1, and 3 DPA. *IAA7* (Gorai.009G132200), *ARF16* (Gorai.011G238900), *ethylene receptor* (Gorai.002G038300), *MYB transcription factor* (Gorai.013G196800), *tcp transcription factor* (Gorai.012G166500), and *Endo-β-glucanase* (Gorai.007G126900). All RT-qPCR analyses were performed with three biological replications at each time point with five technical replications. The error bars represent SD.

Class 2 DEGs, “DEGs more highly expressed in fiberless mutant ovules than in WT ovules at 3 DPA”, were equivalently expressed in the ovules of WT and the three fiberless mutants from -3 to 1 DPA, but their expression levels were noticeably different at 3 DPA. The expression levels of *IAA7*, *ARF16*, *ethylene receptor*, *MYB transcription factor*, *tcp transcription factor*, and *endo-β-glucanase* were all higher in the fiberless mutant ovules at 3 DPA than in the WT ovules containing elongating fibers (Fig 7). In addition to the six genes presented in Fig 7, many other genes including *programmed cell death protein* (Gorai.007G244700), *auxin-response gh3-like protein* (Gorai.005G234200), *zinc finger transcription factor* (Gorai.011G072500), *serine carboxylase peptidase 2* (Gorai.006G103600), *heat shock Hsp 101 chaperone* (Gorai.012G003700), and *heat shock protein-associated protein* (Gorai.007G021300) belong to Class 2 DEGs.

**Fig 8:**
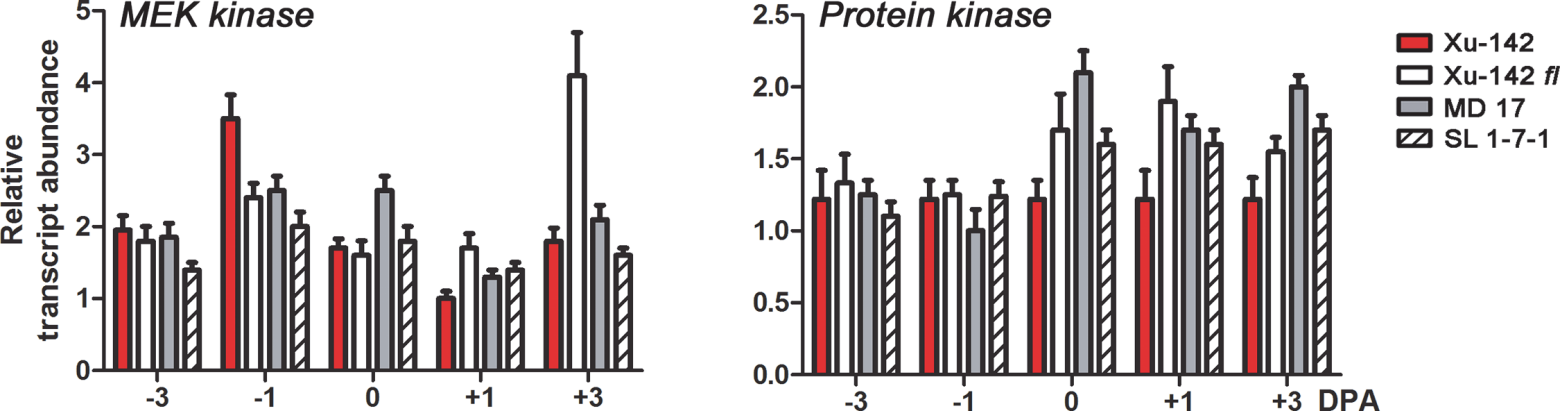
Expression patterns of Class 3 DEGs, genes constitutively expressed in wild type and the fiberless mutants. Transcript levels were measured by RT-qPCR from a WT (Xu-142) and three fiberless mutants (Xu-142 *fl*, MD 17, and SL 1-7-1) that were harvested on -3, -1, 0, 1, and 3 DPA. *MEK kinase* (Gorai.012G034500), and *protein kinase* (Gorai.008G294700). All RT-qPCR analyses were performed with three biological replications at each time point with five technical replications. The error bars represent the SD.

Class 3 DEGs, “DEGs constitutively expressed in WT and mutants”, were not differentially expressed in WT ovules and fiberless mutant ovules although these genes were differentially regulated in cultured ovules by exogenous phytohormone treatment that triggered fiber initial differentiation. *MEK kinase* and *protein kinase* that are involved in the mitogenic activated protein kinase (MAPK) signaling pathway belong to Class 3 DEGs (Fig 8).

## Discussion

### Cotton fiber differentiation in pre-anthesis ovules is regulated by an exogenous application of phytohormones

To understand the molecular mechanisms regulating cotton fiber differentiation from ovular epidermal cells, we took advantage of the cotton ovule culture technique. Cotton ovule culture methods have been optimized, and many groups have found that fiber development and cell wall composition *in vitro* closely mimic fiber formation *in planta* [33,49]. Despite either gibberellic acid (GA) or auxin can differentiate fiber initials from unfertilized ovules, abnormal fiber development [50] or inefficient differentiation of fiber initials [51] were observed when unfertilized ovules were cultured with either phytohormone. Thus, the optimized concentrations of two phytohormones, indole-3-acetic acid (IAA) and GA producing normal fiber development were used for differentiating fiber initials from unfertilized cotton ovules [33,49]. Due to the biochemical and developmental similarities between the fibers cultured with IAA and GA *in vitro* and the fibers grown *in planta*, cotton ovule culture has been used to identify regulatory factors affecting fiber elongation by brassinosteroids [52,53], ethylene [42], phytosulfokine peptide hormone, extracellular ATP levels [54], and reactive oxygen species [55]. Although the same exogenous phytohormone treatments promote the differentiation of fiber initials on pre-anthesis (-3 to -1 DPA) cultured ovules, the culture technique has not been used for determining transcriptome profiles during the fiber initiation stage of fiber development.

To study cotton fiber initial differentiation, we started all ovule cultures with -1 DPA cotton ovules harvested before 0900 with no fiber initials on the surface (Fig 1). Exogenous application of auxin and GA enabled the differentiation of fiber initials from the -1 DPA ovular epidermal cells within 24 hours, whereas the control cultures without the phytohormones did not differentiate fiber initials (Fig 2). Thus, -1 DPA ovules may be more enriched in transcripts involved in the differentiation of fiber initials than the 0 and 1 DPA ovules that were previously used for other transcriptome analyses [14,15,16]. Our conclusion is also supported by biochemical analyses revealing that fiber cell fate determination occurs prior to the formation of morphologically visible fiber initials [5,13,56].

### *IAA29*, the most up-regulated gene in pre-anthesis cotton ovules by exogenous phytohormones, is co-regulated *in planta* with *MYB25-like*, a gene involved in cotton fiber differentiation

Using the cultured ovules, transcriptome profiles were monitored frequently (1, 3, 6 and 12 h) after the addition of auxin and GA, phytohormones that together triggered the differentiation of fiber initials. Previously reported transcriptome analyses [12,13,14,15,16,34] were mostly conducted at one time point or multiple time points over several days, making it difficult to determine the cascade of phytohormone signaling and networks that affect the temporal regulation of genes involved in the differentiation of fiber initials (Figs 1 and 2).

The transcript abundance of both *MYB25-like* and *HD-1*, reportedly involved in the differentiation of fiber initials, was up-regulated around one or two days pre-anthesis [27,28]. In our study, striking differences in *MYB25-like* transcript abundance between WT and three fiberless mutants were detected in -1 DPA ovules (Fig 7). Thus, the comparison of transcript levels in cotton ovules at -1 DPA between WT and three genetically different fiberless mutants provided us with a means to identify genes that are co-regulated with *MYB25-like* and *HD-1*. Among the transcription factors identified by GO enrichment analysis (Table 1), genes involved in auxin signaling were temporally up-regulated during fiber initial differentiation *in vitro*. A pattern of expression similar to *MYB25-like* [26] was evident for *INDOLE-3-ACETIC ACID INDUCIBLE 29* (*IAA29*), a Class 1 DEG and the most highly up-regulated phytohormone-induced gene after 1 h phytohormone treatment in the cultured ovules. Like *MYB25-like*, *IAA29* was substantially up-regulated *in planta* in WT ovules at -1 DPA over ovules of the same age from the fiberless mutants (Figs 6 and 7). In contrast to *MYB25- like* whose expression levels decreased in WT ovules at 1 DPA, *IAA29* decreased in WT ovules at 0 DPA. Walford et al [27] previously showed that transcript abundance of an upstream *MYB25- like* gene diminished on earlier than that of a downstream *MYB109* gene involved in fiber development. IAA29 (AT4G32280) belongs to the auxin/indole-3-acetic acid-responsive (AUX/IAA) protein family of genes that play crucial roles in auxin regulation of *Arabidopsis* development, such uni-dimensional cell growth, cell wall loosening, cell proliferation, cell expansion, nucleosome organization, and DNA-protein complex formation [57,58]. The Aux/IAA proteins interact with ARFs to regulate auxin-responsive genes by controlling the activity of ARFs [59,60]. Among twenty-nine Aux/IAA genes identified in *Arabidopsis*, auxin-inducible *IAA29* is involved in clock-controlled plant developmental events such as photoperiodic control of flowering time and hypocotyl elongation [61]. Transgenic cotton lines containing higher auxin levels in the ovule epidermis at anthesis from the overexpression of *iaaM*, an IAA biosynthetic gene, had higher densities of fiber initials [11]. Thus, it would be interesting to test if *IAA29* may be an upstream gene of *MYB25-like* that controls fiber initial differentiation. The expression patterns of nine *Aux/IAA* transcripts (*GhAux1* to *GhAux9*) from *G*. *hirsutum* were previously compared between WT and fiberless mutants [62]. In ovules at the initiation phase of fiber development (-3 to 1 DPA), most *GhAux* genes were down-regulated in WT as compared with the fiberless mutants unlike *IAA29* that was up-regulated in WT as compared with the fiberless mutants (Fig 6). Our results showing that the auxin signaling pathway is mainly involved in fiber differentiation are consistent with previous reports that auxin is required for fiber development from unfertilized cotton ovules but not from fertilized ovules that already have fiber initials [33,35]. Numerous AUX/IAA transcriptional regulators of the auxin signal transduction pathway are known to be involved in the differentiation of root hair cells in *Arabidopsis* [30], and GhTCP14 functions in auxin-mediated epidermal cell differentiation and elongation during cotton fiber development [41].

Other Class 2 DEGs involved in auxin signaling pathways such as AUX/IAA genes (*IAA 4*, *7*, *11*, and *16*), *ARF16*, *auxin-response gh3-like protein*, and a *TCP transcription factor* were up-regulated *in vitro* by the exogenous auxin and GA within 1 h (Table 1 and Fig 4). During fiber initiation (-3 to 1 DPA) *in planta*, there was little difference in the expression levels of these Class 2 DEGs between WT and three fiberless mutants, whereas they decreased as fiber elongated in 3 DPA WT ovules unlike the corresponding fiberless mutant ovules in which their expressions were maintained (Fig 7). As results, they may be involved in ovule development rather than fiber initial differentiation.

Several Class 3 DEGs, such as *MEK kinase* and *protein kinase*, that are involved in the auxin-induced MAP kinase signaling pathway responding to wounding and root development in *Arabidopsis* [63,64] were also up-regulated *in vitr*o by the exogenously applied phytohormones. Since these Class 3 DEGs were equivalently expressed *in planta* between WT and fiberless mutants, they are unlikely to be involved directly in fiber initial differentiation; however, we do not rule out a potential involvement of the MAP kinase pathway in early fiber and/or ovule development since multiple genes involved in jasmonic acid signaling pathways for defense responses were also identified by our transcriptome analysis (Table 2). Exogenous application of hydrogen peroxide is known to be involved in signaling pathways responding to defense and wound-facilitated fiber initiation in another fiberless mutant, XinFLM [65]. Thus, further research is required to conclude if and how the auxin-induced MAP kinase signaling pathway and jasmonic acid signaling pathways affect differentiation of cotton fiber initials.

### Crosstalk among the auxin, ethylene, and ABA signaling pathways contributes to cotton fiber differentiation from epidermal cells

In addition to *IAA29* in the auxin signaling pathway, the expression patterns of *ACC oxidase* involved in ethylene production and *ABA 8'-hydroxylase* involved in ABA catabolism were co-regulated both *in vitro* and *in planta* with *MYB25-like* and *GhHD-1* that are indispensible for cotton fiber differentiation (Table 2, Figs 4 and 6). Unlike *MYB25-like* and *IAA29* that were differentially expressed in the ovules from both WT and fiberless mutants, *ACC oxidase* and *ABA 8’-hydroxylase* were mostly found in WT ovules but little in the ovules from the fiberless mutants (Fig 6). In addition to the tissue specific expression, their temporal expression patterns at -1 DPA just before differentiating fiber initials in WT ovules also suggest potential involvement in fiber initiation process.

ACC oxidase (EC 1.14.17.4) catalyses the final step in the biosynthesis of the phytohormone ethylene from 1-aminocyclopropane-1-carboxylate (ACC) as a substrate. Synergistic interactions between auxin and ethylene-dependent phenomena have been reported from *Arabidopsis* [42,66,67]. In *Arabidopsis* root development, auxin is a positive regulator for ethylene-mediated response [66], and normal auxin signaling is required for a proper ethylene response in *Arabidopsis* hypocotyls [67]. Identical up-regulation of both *IAA29* for auxin signaling and *ACC oxidase* for ethylene signaling during the fiber initiation phase (Fig 6) suggests that synergistic interactions between auxin and ethylene may be necessary for the differentiation of fiber initials.

Abscisic acid (ABA) 8'-hydroxylase is also known as a cytochrome P450 (CYP707A) that catalyzes the first step in the oxidative inactivation of ABA [43,68]. The ABA 8'-hydroxylase converts ABA to 8'-hydroxy-ABA that is unstable and isomerizes to form phaseic acid. Therefore, ABA 8'-hydroxylase can regulate physiologically active ABA levels during plant developmental process. ABA is known to inhibit fiber initiation in cultured ovules [69]. The elevated levels of *ABA 8'-hydroxylase* in WT ovules (-1 DPA) compared with the fiberless mutant ovules should reduce the level of ABA that could inhibit fiber development, thereby stimulating the differentiation of cotton fiber initials (Figs 4 & 6).

## Conclusions

**Fig 9:**
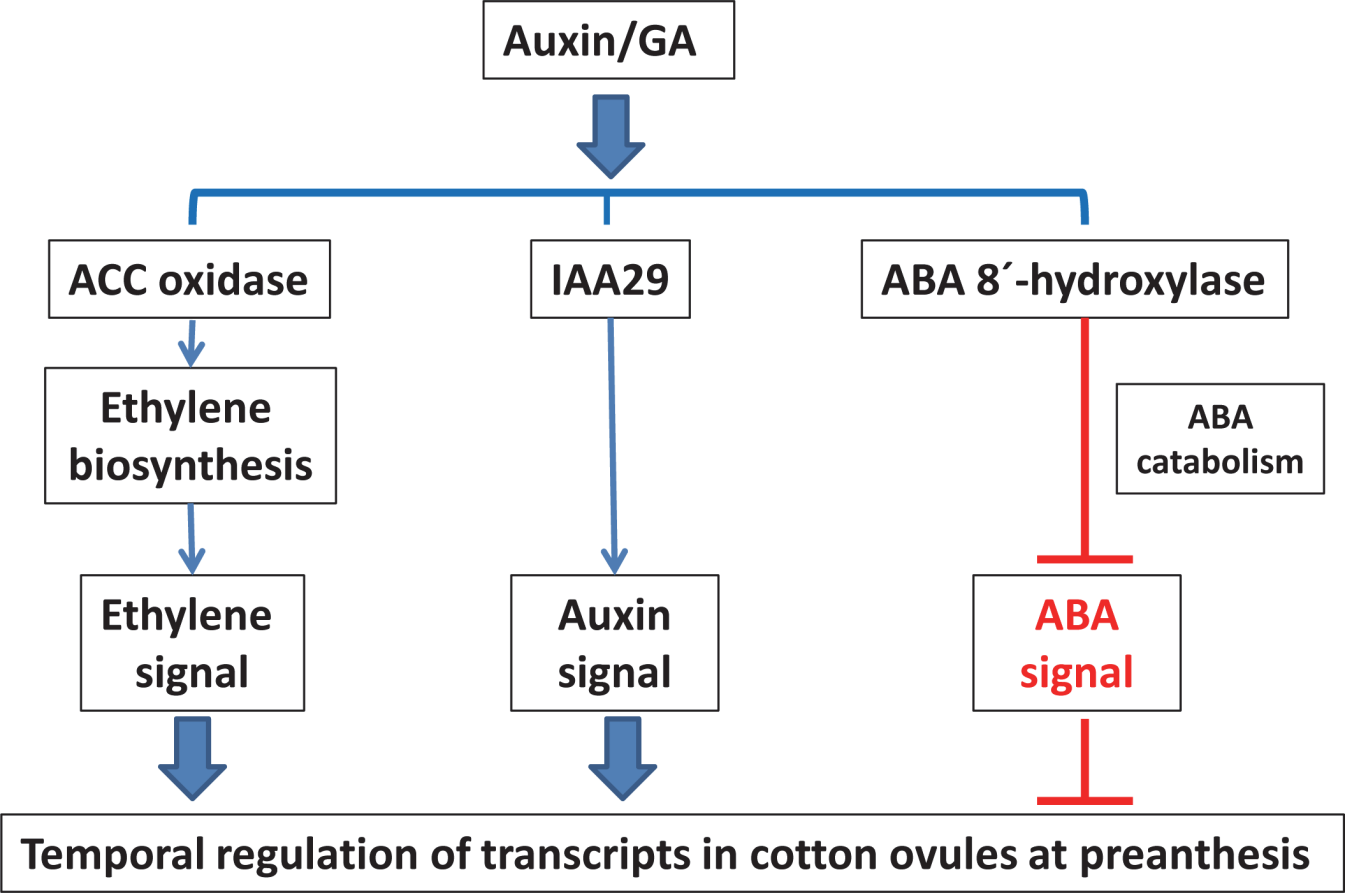
Genes co-regulated *in vitro* and *in planta* with *MYB25-like*.

Technical difficulties in monitoring the rapid process of fiber initial differentiation in ovaries and extracting RNAs from very short and small fiber initials without contamination with epidermal tissues has hindered the progress of cotton fiber initiation research. Pioneering investigations using the fiberless cotton mutants [16] and fiber initials extracted by a laser capture microdissection technique [14] produced transcriptome profiles from fiber initials differentiating on 0 DPA cotton ovules. Despite these efforts, recent functional analyses of the candidate genes identified by these earlier transcriptome studies showed that the candidate genes are more likely to be involved in fiber elongation than in fiber initial differentiation. To identify putative genes involved in fiber initial differentiation and understand the molecular mechanisms of differentiating fiber initials, we improved the conditions for the comparative transcriptome expression analyses to enrich for transcripts involved in cotton fiber initial differentiation *in vitro* and *in planta*. Our study identified genes that are co-regulated with *MYB25-like* and *HD-1*, two genes previously identified as key transcriptional factors responsible for fiber initial differentiation (Fig 9). We have identified candidate genes for fiber initiation whose contribution must be more completely analyzed to discover how phytohormonal signaling pathways and crosstalk regulate the temporal expression of genes responsible for the differentiation of fiber initials *in vitro* and *in planta*.

## Supporting Information

S1 Fig Scanning microscopic images of a fiberless mutant. (TIF)

S1 File Expression data for significantly regulated genes. (XLSX)

S1 Table Primer sequences for RT-qPCR. (DOCX)
