# Supplementary figures and images for: Phytohormonal Networks Promote Differentiation of Fiber Initials on Pre-Anthesis Cotton Ovules Grown In Vitro and In Planta
Source: PLoS One. 2015 Apr 30;10(4):e0125046. doi: 10.1371/journal.pone.0125046 (PMC4415818; doi:10.1371/journal.pone.0125046)

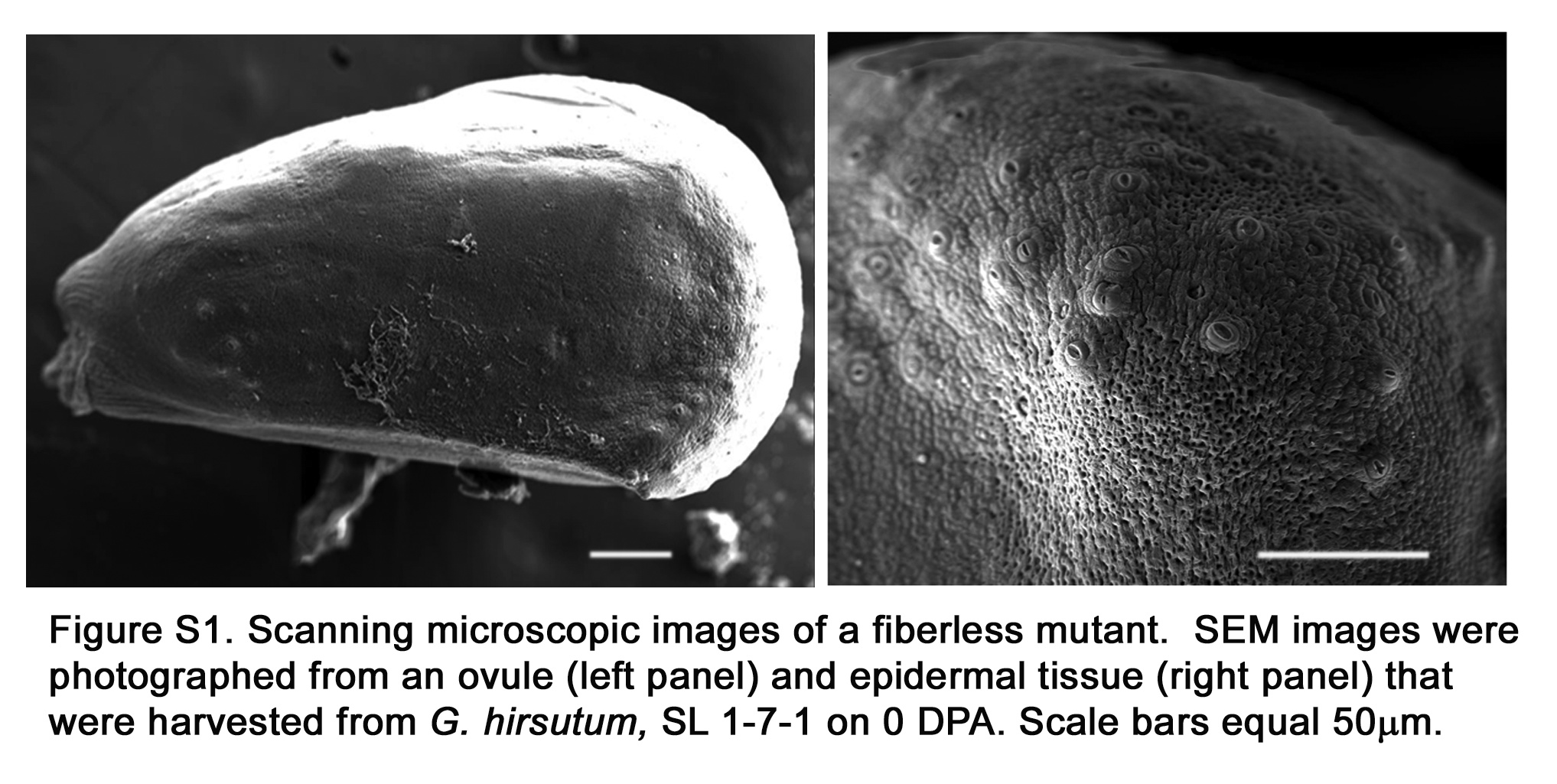

Supplement: S1 Fig — (TIF) [file pone.0125046.s001.tif]
