# Supplementary material for: Phytohormonal Networks Promote Differentiation of Fiber Initials on Pre-Anthesis Cotton Ovules Grown In Vitro and In Planta
Source: PLoS One. 2015 Apr 30;10(4):e0125046. doi: 10.1371/journal.pone.0125046 (PMC4415818; doi:10.1371/journal.pone.0125046)
Supplement: S1 Table — (DOCX) [file pone.0125046.s003.docx]

**S1 Table. Primer sequences for RT-qPCR**

| No. | Annotation | *G. raimondii* ID | Forward primer 5′to 3′ | Reverse primer 5′to 3′ | |
| --- | --- | --- | --- | --- | --- |
| 1 | *IAA29* | Gorai.001G043900 | GAAGGTAAAGATGGAAGGAAATGC | | AAAGCTCTTTTGCCCATTCTCA |
| 2 | *auxin-responsive GH3-like protein* | Gorai.005G234200 | TCCATTTCGTGAGGAGGAAGA | | GCTTTTTGTAACTCGGCTTCGT |
| 3 | *auxin response factor 16* | Gorai.011G238900 | TGTTTGGTCAACTCATTTTCTGTGA | | CCCATCTGATGAACTGTTTCCA |
| 4 | *protein kinase* | Gorai.008G294700 | TGGCTCCCGAGGTTCTCA | | CGAAGCTGTACACATCGCATTT |
| 5 | *TCP family transcription factor* | Gorai.012G166500 | CATCCGAACCCCAGATATGG | | TGTAAGGGTGCTTGCAATGTG |
| 6 | *myb transcription factor* | Gorai.013G196800 | CCATCTGAAGAGGAAGCTAAGGAA | | TCACCCGCCATTGAAGGA |
| 7 | *ABA 8'-hydroxylase* | Gorai.004G177200 | CGTTTCCAACATCGAATCCA | | TCAGCCTGCCTTCCAATGAC |
| 8 | *ethylene receptor* | Gorai.002G038300 | TGGGTCGAACTTTGGCACTAG | | GAAAGTTGGAGCTCAAGTCCAGTAC |
| 9 | *endo-β-glucanase* | Gorai.007G126900 | GGGCTCCTGTTTAAGATGAGTGA | | AAGCAGGAAGGAAGTGGAGGTA |
| 10 | *IAA 16* | Gorai.009G132200 | TCCCGTTTCAAAGACACAAGTG | | AACTCTTCTTTCCTCTCTTTGCAAA |
| 11 | *MEK kinase (MAP3Ka)* | Gorai.012G034500 | GGCTACAACCTCAAGAGCACTGA | | GGGCAACGATGTGATTGTTCT |
| 12 | *Auxin induced gene, IAA11* | Gorai.007G150000 | GCTGATGTCACAGCTAAGATCAAGA | | AGGAGGCCACCCCACAA |
| 13 | *GhHD-1 (leucine-rich repeat LRR protein)* | Gorai.013G167800 | GCTGAAGTTGTTGGATGTGTCTTT | | AAGAAGTTGCCGAGCCAATC |
| 14 | *heat shock protein (Hsp 101) chaperone* | Gorai.006G011800 | GCTCATGGTGATGTATTTAATGTTTTC | | GCTCACGGTACGACCTTGTGA |
| 15 | *lipid transfer protein* | Gorai.008G057100 | TGCTGTTTTCGGGTGAAAGTC | | GCTCCACGGGATTACATGTCA |
| 16 | *GhMyb25-like* | Gorai.008G179600 | GAGAAATCGAGCCAAGTTGC | | GATCCCCAGAATCACAAACC |
| 17 | *ACC oxidase* | Gorai.001G011100 | GGGAATAGGCTTTCCATTGCT | | GGGAATAGGCTTTCCATTGCT |
| 18 | *18S rRNA* | U42827 | CGTCCCTGCCCTTTGTACA | | AACACTTCACCGGACCATTCA |
| 19 | ubiquitin-conjugating protein | AI730710 | CGGAAAGAGGTGAAGATGTCAAC | | GGATCTTGCTGCAACCTCTTAAA |
| 20 | α-tubulin 4 | AF106570 | GATCTCGCTGCCCTGGAA | | ACCAGACTCAGCGCCAACTT |
